# The Early Antibody-Dependent Cell-Mediated Cytotoxicity Response Is Associated With Lower Viral Set Point in Individuals With Primary HIV Infection

**DOI:** 10.3389/fimmu.2018.02322

**Published:** 2018-10-09

**Authors:** Xi Chen, Meilin Lin, Shi Qian, Zining Zhang, Yajing Fu, Junjie Xu, Xiaoxu Han, Haibo Ding, Tao Dong, Hong Shang, Yongjun Jiang

**Affiliations:** ^1^Key Laboratory of AIDS Immunology of National Health and Family Planning Commission, Department of Laboratory Medicine, The First Affiliated Hospital of China Medical University, Shenyang, China; ^2^Key Laboratory of AIDS Immunology of Liaoning Province, The First Affiliated Hospital of China Medical University, Shenyang, China; ^3^Key Laboratory of AIDS Immunology, Chinese Academy of Medical Sciences, Shenyang, China; ^4^Collaborative Innovation Center for Diagnosis and Treatment of Infectious Diseases, Hangzhou, China; ^5^Affiliated Hospital of Hebei University, Baoding, China; ^6^MRC Human Immunology Unit, Radcliffe Department of Medicine, Oxford University, Oxford, United Kingdom

**Keywords:** human immunodeficiency virus, natural killer cells, primary HIV infection, antibody-dependent cell-mediated cytotoxicity, epitopes

## Abstract

Antibody-dependent cell-mediated cytotoxicity (ADCC) is an immune response largely mediated by natural killer (NK) cells that can lyse target cells and combat tumors and viral infections. However, the role of ADCC in response to primary HIV infection is poorly understood. In the present study, we explored the ADCC response and evaluated its characteristics in 85 HIV-infected individuals, including 42 with primary infections. Our results showed that ADCC occurs during acute infection, and the earliest ADCC response to a single peptide was detected at 52 days. Primary HIV-infected individuals exhibiting ADCC responses had lower viral set points than those with no ADCC response, and functional analyses demonstrated that the ADCC response could significantly inhibit viral infection during primary HIV infection. HIV epitopes that provoked the ADCC response were determined and three relatively conserved epitopes (HNVWATYACVPTDPNPQE, TSVIKQACPKISFDPIPI, and VVSTQLLLNGSLAEEEII) from the surface of the three-dimensional structure of the HIV Env protein were identified. Overall, our data indicate that ADCC responses may be significant for the control of HIV from an early stage during infection. These findings merit further investigation and will facilitate improvements in vaccines or therapeutic interventions against HIV infection.

## Introduction

Human immunodeficiency virus (HIV) can infect CD4^+^ T cells (1) and cannot be efficiently cleared by the immune system (2). To date, there are no sufficient medicines to cure HIV and no vaccine to prevent it. Thailand RV144, the only effective HIV vaccine, partially protects against HIV-1 (31%), possibly by stimulation of antibody-dependent cell-mediated cytotoxicity (ADCC) (3–5).

The ADCC immune response is largely mediated by natural killer (NK) cells. In the presence of a tumor or virus, the Fab domains of IgG antibodies bind specifically to antigens expressed on the surface of the tumor or virus-infected target cells, while the Fc domain of IgG antibodies bind to NK cell CD16 Fc receptors, triggering the release of gamma interferon (IFN-γ), perforin, tumor necrosis factor alpha (TNF-α), granzyme B, interleukin-1 (IL-1), and granulocyte-macrophage colony-stimulating factor (GM-CSF) (6). There is increasing evidence that ADCC response can influence the replication of HIV, and more robust ADCC responses are associated with slower disease progression and lower viral loads (7–10).

Acute HIV infection is a critical stage which determines subsequent disease progression. During primary HIV infection (PHI), viral loads peak, subsequently reducing to more stable levels, referred to as the viral set point (11). The level of the viral set point may predict both the possibility of HIV transmission during chronic HIV infection (CHI) and disease progression; therefore, the relationship between ADCC response during PHI and viral set point warrants further study.

Studies in rhesus macaques showed that the ADCC response emerged after 3 weeks and viral set point in early infection determined the speed of disease progression (12). The magnitude of the ADCC response is also reported to be negatively correlated with viral set point in acute phase of simian immunodeficiency virus (SIV) infection (13). Thus, events during PHI may be important in determining viral set point and disease progression; however, the role of ADCC responses in human PHI is not well understood; in particular, the timing of the initiation of ADCC responses and the HIV epitopes recognized as part of this process have yet to be determined.

In this study, we explored the NK cell-mediated ADCC response in individuals with PHI and found that initial ADCC response to a single Env protein peptide motif was at 52 days after HIV infection. Our results indicated that PHI who had positive ADCC responses had lower viral set point. We also found that ADCC response could suppress viral infection *in vitro*. Moreover, we identified HIV epitopes that provoked ADCC responses and analyzed their molecular locations. Our findings provide reference data for use in future preclinical vaccination studies and antibody development.

## Materials and methods

### Study subjects

Eighty-five HIV-infected subjects were recruited from the First Affiliated Hospital of China Medical University. The clinical characteristics of infected individuals are summarized in Table 1. Subjects were divided into three groups according to infection phase: (1) individuals with PHI; (2) those with CHI; and (3) patients receiving highly active antiretroviral therapy (HAART). The PHI group, who were from a high-risk HIV-negative cohort who were tested every 4–16 weeks for HIV antibodies and antigen, or HIV pooled nucleic acid, were defined as those with HIV infection-positive results within 180 days and not receiving HAART. The CHI group was defined as those with HIV infection beyond 180 days and not receiving HAART. The HAART group comprised patients receiving HAART after HIV infection. Enrolled subjects were provided written informed consent and the relevant human research ethics committees approved this study.

**Table 1:** Clinical characteristics of subjects enrolled in this study.

**Characteristics**	**PHI group**	**CHI group**	**HAART group**
Subject no.	42	22	21
Age (years, mean ± SD)	34 ± 5	32 ± 7	33 ± 6
Male (No, %)	42 (100%)	22 (100%)	21 (100%)
CD4 (cells/μL, mean ± SD)	468 ± 197	394 ± 194	401 ± 98
Viral load (Lg copies/mL, mean ± SD)	4.67 ± 1.14	4.1 ± 0.8	1.26 ± 1.49
Estimated infection time (days, mean ± SD)	70 ± 43	662 ± 463	1426 ± 2069
Average estimated infection time with ADCC positive response (days)	76.9	570	1847

The estimated time of HIV infection was a time point between the last time with negative HIV test results and the first follow-up point with 3^rd^ ELISA (-) NAAT (+) or 3^rd^ ELISA (+) NAAT (+) results. Two criteria were used to estimate the infection time: first, if the patient could clearly recall the time of high-risk exposure, that time point was the estimated infection time; second, if not, the midpoint between the last time point of negative NAAT results and the first time point of positive NAAT results was the estimated infection time. The estimation of all patients' infection time referred to the Fiebig stage (14).

### Screening tests of HIV infection

The preliminary screening tests were carried out according to the instructions using the HIV Antibody Diagnostic Kits (the third generation ELISA test, BioMerieux, Dutch). If the preliminary screening results were positive, we performed rescreening tests using the HIV 1+2 Colloidal Selenium Rapid Test Kits (Abbott Laboratories, USA).

### Diagnosis tests of HIV infection

The western assays were performed according to the instructions using the HIV 1+2 Antibody Detection Kit (MP Biomedical Asia Pacific Private Ltd., Singapore).

### Pooled nucleic acid amplification testing (NAAT)

NAAT of Mixed samples were performed on Roche Molecular Systems (USA), and if the NAAT results were positive then the mixed pools were split step by step to find the positive samples.

### HIV peptides

Based on the Env consensus sequences of the CRF01-AE subtype from local HPI subjects with PHI, 106 Env 18-mer overlapping peptides (overlapping by 10 amino acids) were synthesized by CL. Bio-Scientific Co. Ltd. (Xi'an, China). The peptides were divided into three pools, each containing 27–42 peptides (Pool 1: peptides 1–36 from gp120, C1-V1-V2-C2; Pool 2: peptides 37–64 from gp120 V3-C3-V4-C4-V5; Pool 3: peptides 65–106 from gp41).

### Measurement of the NK cell-mediated ADCC response

**Figure 1:**
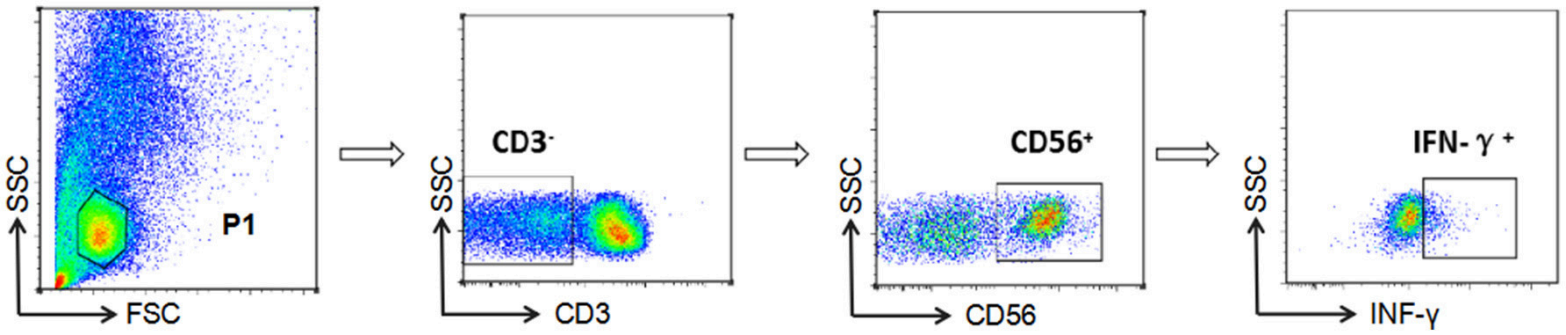
Gating strategy for identification of NK cell-mediated ADCC responses. NK cells were identified according to their surface phenotype (CD3^−^CD56^+^), and ADCC responses were determined from the percentage of cells exhibiting intracellular INF-γ expression.

NK cell-mediated ADCC responses were evaluated by measurement of INF-γ expression by ADCC-activated NK cells, as described previously (6, 15, 16). In brief, whole blood (150 μL) from a HIV-negative donor and serum (50 μL) from a HIV-positive patient were incubated at 37°C with either HIV peptide pools (20 μg/mL) or dimethyl-sulfoxide (DMSO, Sigma) for 5 h in the presence of Brefeldin-A (BD Biosciences) and Monensin (GolgiStop, BD Biosciences). Following incubation, red blood cells were lysed using lysing solution (2 mL) and white blood cells were stained with anti-CD3 and anti-CD56 antibodies (BD Biosciences), then fixed and permeabilized using a Fixation/Permeabilization Solution Kit (BD Biosciences), and intracellular IFN-γ was stained (BD Biosciences). Phorbol 12-myristate 13-acetate (PMA, 50 ng/mL) was used as a positive control and DMSO (1 mg/mL) as a negative control. Data were collected using an Aria II flow cytometer (BD Biosciences) and analysis performed with FSCDiva software. INF-γ production by NK cells was measured to evaluate the magnitude of the ADCC response (Figure 1). Two criteria were required for a positive ADCC response to be recorded: first, the percentage of IFN-γ^+^ NK cells should be more than three times that of control samples incubated with DMSO; second, the percentage of IFN-γ^+^ NK cells should be more than the mean value plus two standard deviations calculated using data from 10 separate readings from serum specimens from negative donors and estimated for each of the peptide pools (17).

### Detection of inhibition of viral infection via ADCC response

To detect whether the ADCC response can influence viral infection, primary CD4^+^ T and NK cells were sorted from HIV-negative donors. NK cells were stimulated with IL-12 (10 ng/mL, R&D), IL-15 (50 ng/mL, R&D), and IL-18 (100 ng/mL, R&D) for 3 days. CD4^+^T cells were stimulated with PHA (5 μg/mL, Sigma) and IL-2 (20 U/mL, Roche) for 1 day, and were infected with the primary HIV isolate for 2 days. The primary isolate (R5 tropic, subtype CRF01_AE) had been isolated from the PBMCs of the MSM (men who have sex with men) at 63 days of HIV infection in our cohort. The primary isolate was generated in PHA and IL-2 stimulated PBMCs, which has been described previously (18). After 5 days, supernatant from infected cells was positive for p24 antigen by ELISA; after 22 days, p24 antigen level of supernatant reached to peak value (16 ng/mL). The determination of the tropism was performed in Ghost-CCR5 and Ghost-CXCR4 cells by fluorescence microscope and flow cytometry. Then CD4^+^ T cells were plated in 500 μl of Roswell Park Memorial Institute (RPMI)-1640, 10% fetal bovine serum (FBS), and IL-2 (20 U/mL), and co-cultured with autologous NK cells (effector:target ratio = 1:5) with serum samples from the PHI group (with/without ADCC response) or HIV-negative donors for 3 days. Supernatants were collected from each culture and tested for the HIV-1 P24 antigen by enzyme linked immunosorbent assay (ELISA) kits (QuantoBio, Beijing, China).

### Blockade of anti-CD16 during the ADCC response

NK cells were sorted from HIV-negative donors and stimulated with IL-12 (10 ng/mL), IL-15 (50 ng/mL), and IL-18 (100 ng/mL) for 2 days. Then, NK cells were incubated with purified anti-human CD16 blocking antibody (50 μg/mL, BioLegend) or purified mouse IgG1, κ isotype antibody (50 ug/mL, BioLegend) for 1 day. Infected primary CD4^+^ T cells were co-cultured with autologous NK cells (effector:target ratio = 1:5) and serum samples from the PHI group (with/without ADCC response) or HIV-negative donors for 3 days. Supernatants were collected and tested for HIV-1 P24 antigen by ELISA.

### Determination of epitopes recognized in ADCC responses

To identify individual reactive peptides, each peptide pool was further divided into 11, 12, or 13 small pools according to five by six, five by seven, or six by seven matrix formats (19). Measurement of INF-γ expression by ADCC-activated NK cells was performed as described previously (6, 15, 16).

### Measurement of HIV viral load

Reverse transcription-polymerase chain reaction (RT-PCR) was used to test plasma HIV viral loads (Roche Molecular Systems, USA). The limit of detection was 20–75000 copies/mL. For the PHI group, viral loads at 70–120 days after infection were defined as the viral set point.

### Calculation of epitope conservation

Epitope conservation was determined by calculating epitope prevalence and Shannon entropy scores. For epitope prevalence scores, peptides present in ≥60% of 37 Env sequences from the CRF01-AE subtype from local HIV infected subjects were considered conserved epitopes (20). Shannon entropy scoring is a universal method for determining conservation that predicts levels of uncertainty (21). Entropy values of local Env consensus sequences from the CRF01-AE subtype were calculated using HIV databases. Epitopes with entropy scores lower than the 20th percentile of all distributions were defined as conserved (22).

### Spatial conformation of conserved epitopes

The spatial structure of a representative Env sequence from the CRF01-AE subtype was simulated using SWISS-MODEL. Synthesized diagrams were presented in PyMOL and the conserved epitopes we found are highlighted.

### Statistical analysis

Statistical analyses were performed using GraphPad Prism 6.0 software (GraphPad software, USA). The non-parametric Mann–Whitney U test was used to compare between-group distributions. Associations between two groups were determined by the Spearman's rank test. A *p* < 0.05 (two-tailed test) was considered statistically significant.

## Results

### The earliest ADCC response to a single peptide was detected at 52 days

**Figure 2:**
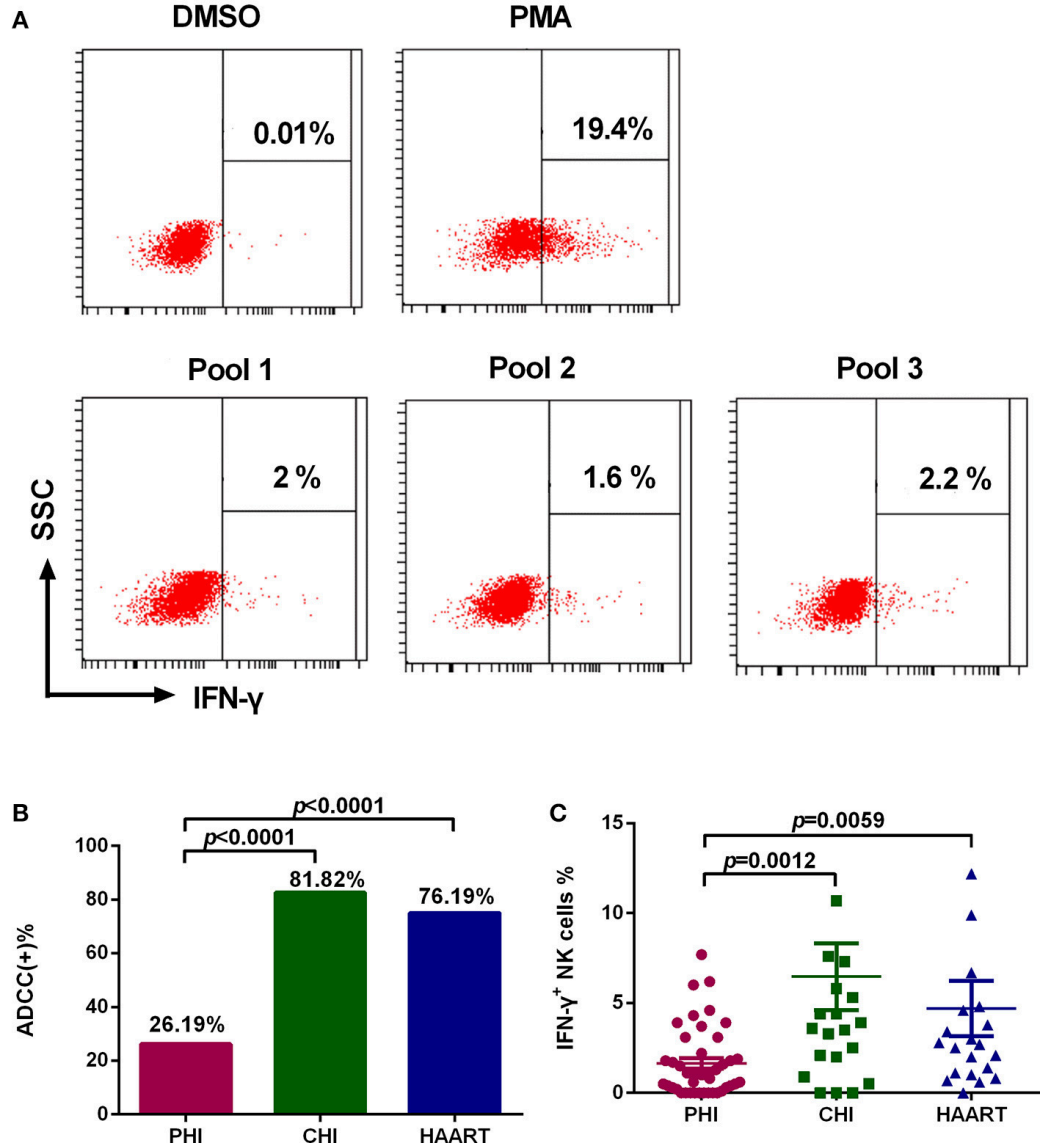
Characteristics of the ADCC response in HIV-infected individuals. **(A)** Flow cytometry plots of IFN-γ production in samples from an individual with PHI at 52 days after infection, incubated with DMSO (negative control), PMA (positive control), and HIV peptide Pools 1, 2, or 3. **(B)** Rates of positive ADCC responses in the PHI (*n* = 42), CHI (*n* = 22), and HAART (*n* = 21) groups. **(C)** Comparison of the percentages of IFN-γ^+^ NK cells during ADCC responses of the PHI (*n* = 42), CHI (*n* = 22), and HAART (*n* = 21) groups.

Among PHI samples (*n* = 42), 11 exhibited ADCC activity against HIV peptide pools, which occurred at average 76.9 days after infection (Table 1), indicating that ADCC antibodies can be produced during primary infection. The earliest ADCC response detected to a single peptide occurred at 52 days after infection (Table 2.1), and the flow cytometry plots of ADCC responses to Pools 1, 2, and 3 are presented in Figure 2A.

**Table 2.1:** Mapping HIV-specific antigen epitopes in PHI group.

**Total responders**	**Peptide no**.	**Sequence**	**Location**	**Estimated infection time**
1	9	HNVWATYACVPTDPNPQE	Env 66 → 83	133
1	12	TENFNMWKNNMVEQMQED	Env 90 → 107	90
1	18	KINATtASNGIGNITDEV	Env 814 → 829	133
1	22	KQKVYALFYKLDIVPIID	Env 169 → 185	150
1	26	TSVIKQACPKISFDPIPI	Env 198 → 215	128
1	29	GYAILKCNDKNFNGTGPC	Env 222 → 239	99
1	32	QCTHGIKPVVSTQLLLNG	Env 246 → 263	99
1	33	VVSTQLLLNGSLAEEEII	Env 254 → 271	52
1	90	FQTPSHHQREPDRPERIE	Env 717 → 734	130
1	91	REPDRPERIEEGGGEQDR	Env 725 → 742	52

### The ADCC response was lower during PHI than that in CHI

The positive ADCC response rate among all subjects included in the study was 52.94% (45 of 85 subjects). The 85 HIV-infected subjects were divided into three groups: PHI, CHI, and HAART. Only 26.19% of individuals in the PHI group exhibited a positive ADCC response, which was significantly lower than in CHI (81.82%, *p* < 0.0001) and HAART (76.19%, *p* < 0.0001) groups. The ADCC positive response rate was also lower in the HAART compared with the CHI group; however, the difference was not significant (Figure 2B). Furthermore, we detected lower IFN-γ expression levels in NK cells in the PHI group compared with the CHI (*p* = 0.0012) and HAART (*p* = 0.0059, Figure 2C) groups.

### ADCC responses reduced the viral set point level in PHI

**Figure 3:**
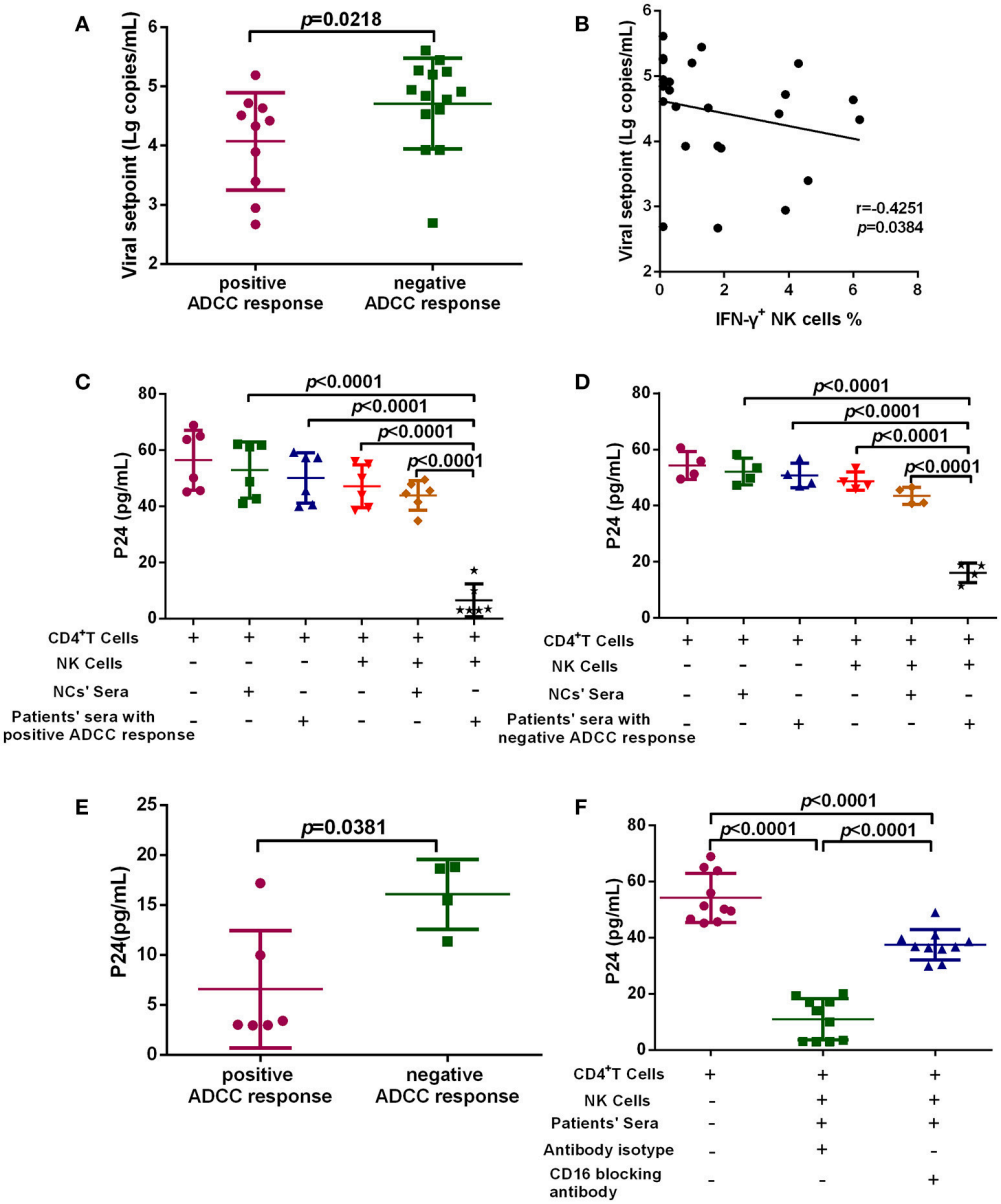
ADCC responses suppress HIV infection and influence viral set point levels. **(A)** Comparison of viral set points of positive (*n* = 10) and negative (*n* = 14) ADCC responses in HIV-infected subjects with viral set point. **(B)** Correlation analysis between the level of ADCC responses and viral set points (*n* = 24). Spearman's rank test was used to correlate the data and *p* < 0.05 was considered significant. **(C)** Inhibition of viral infection by ADCC responses with sera of positive responses (*n* = 6) from PHI group and healthy donors' sera (negative control). **(D)** Inhibition of viral infection by ADCC responses with sera of negative response (*n* = 4) from CHI group and healthy donors' sera (negative control). **(E)** Comparison of the inhibition of viral infection by ADCC response with serum of positive (*n* = 6) and negative (*n* = 4) responses from PHI group. **(F)** The effect of anti-CD16 blocking antibody on ADCC-mediated inhibition of viral infection by evaluation of responses in individuals with PHI (*n* = 10).

To investigate the anti-viral capacity of ADCC responses in PHI, HIV-infected subjects with viral set point were divided into two groups according to their positive or negative ADCC response status, and the viral set points of the two groups were compared. Viral set point values were significantly lower in subjects with positive ADCC responses than in those without ADCC responses (*p* = 0.0218, Figure 3A). We further performed correlation analysis and found that the level of ADCC responses was negatively correlated with viral set points (*r* = −0.4251; *p* = 0.0384, Figure 3B). Moreover, we explored whether the ADCC response in PHI was sufficient to inhibit viral infection. We co-cultured primary CD4^+^ T cells infected with HIV primary isolate, autologous NK cells, and serum from individuals with PHI (with positive ADCC responses) or healthy donors. Initially, we observed that viral infection was suppressed in the culture system including serum from patients with PHI and positive ADCC responses (*p* < 0.0001, Figure 3C). Interestingly, incubation with sera from individuals in PHI group with negative for ADCC response also led to inhibition of viral infection (*p* < 0.0001, Figure 3D); however, the inhibition by PHI sera from those with ADCC responses was stronger compared with that induced by sera negative for ADCC responses (*p* = 0.0381, Figure 3E). To confirm that this inhibition was indeed caused by ADCC, we used an anti-CD16 blocking antibody to restrict the ADCC response, and initially observed that production of HIV-1 P24 antigen in supernatants was enhanced by blockade of CD16 relative to treatment with an isotype control antibody (*p* < 0.0001, Figure 3F). Together, these results suggest that during PHI the ADCC response can reduce the viral set point level and has a critical role in the control of HIV infection.

### Dynamic changes of the ADCC response during HIV infection

**Figure 4:**
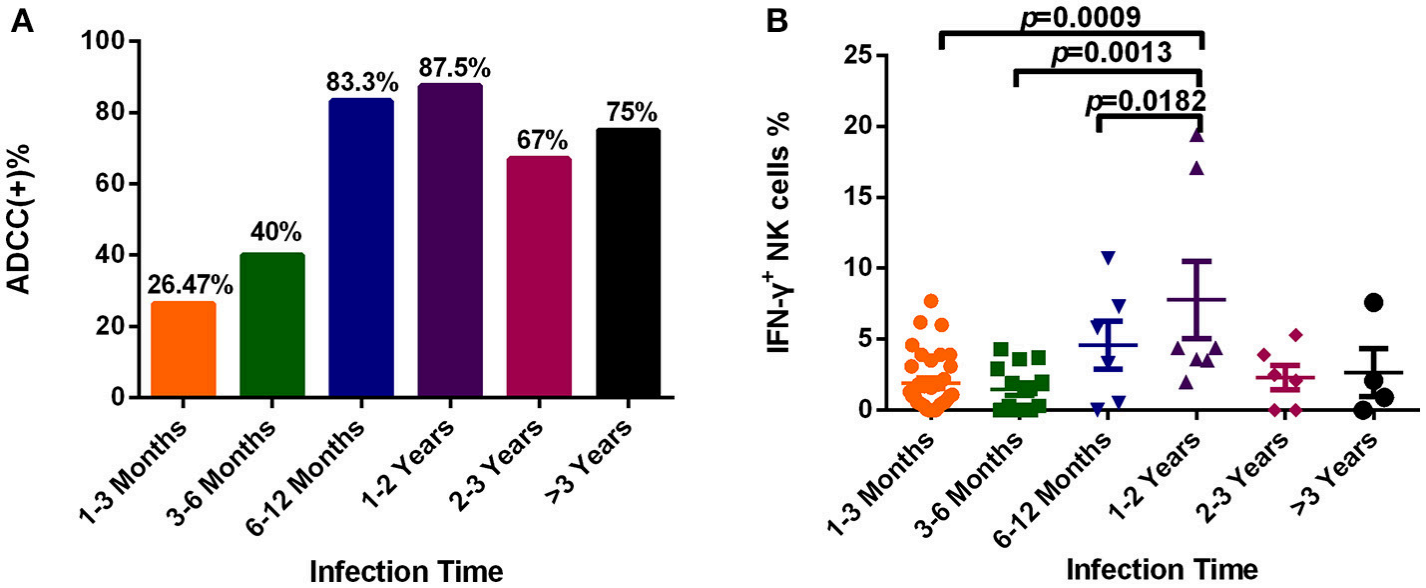
Dynamic changes of the ADCC responses to HIV infection over time**. (A)** Rates of positive ADCC responses at different times during HIV infection (1–3 Months: *n* = 34; 3–6 Months: *n* = 15; 6–12 Months: *n* = 6; 1–2 Years: *n* = 7; 2–3 Years: *n* = 6; >3 Years: *n* = 4). **(B)** Percentages of IFN-γ^+^ NK cells, indicating an ADCC response, at different times during HIV infection (1–3 Months: *n* = 34; 3–6 Months: *n* = 15; 6–12 Months: *n* = 6; 1–2 Years: *n* = 7; 2–3 Years: *n* = 6; >3 Years: *n* = 4).

To explore dynamic changes in the ADCC response, we grouped subjects according to infection time, and detected the positive rate and percentage of IFN-γ^+^ NK cells during the ADCC response. As illustrated in Figure 4A, the positive rate of ADCC response was lower within 6 months of infection, while it rose after 6 months of infection (Figure 4A). Moreover, the percentage of IFN-γ^+^ NK cells associated with the ADCC response during years 1–2 was significantly increased compared with that during acute (within 3 months, *p* = 0.0009) or early (3–6 months, *p* = 0.0013, Figure 4B) infection, and did not elevate further, showing a tendency to reduce from 2 years after infection.

### The strongest ADCC response targeted pool 1 HIV Env peptides

We further analyzed both the positive rate and percentage of IFN-γ^+^ NK cells during ADCC responses to different peptide pools. Pool 1 was frequently recognized by ADCC, leading to the strongest response. In the PHI group with positive ADCC responses, the rate of response to pool 1 (83.3%) was higher compared with those to Pool 2 (50%) and Pool 3 (50%); additionally, the percentage of IFN-γ^+^ NK cells generated in response to Pool 1 was higher (1.83%) compared with those to Pool 2 (1.06%) and Pool 3 (1.09%). In the CHI and HAART groups, among the three pools the positive rates of ADCC responses were the highest to pool 1 (77.8, 87.5%), which was consistent with the percentage of IFN-γ^+^ NK cells generated in response to Pool 1 (4.68, 3.29%). The percentages of IFN-γ^+^ NK cells involved in the ADCC responses to different peptide pools during the acute infection phase were lower than those in the CHI and HAART groups (Table 3).

### Mapping of ADCC responses to HIV epitopes

**Figure 5:**
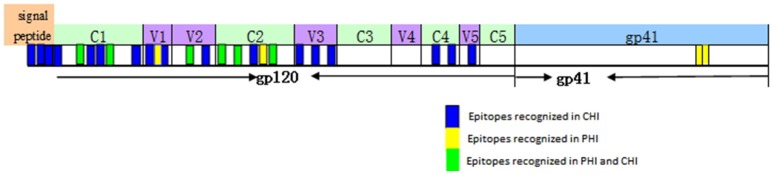
The location of epitopes recognized by ADCC responses in individuals with PHI and CHI. Thirty-three single peptides were identified by analysis of the ADCC responses of individuals with PHI and CHI. Ten single peptides (blue and green), primarily located in the V1/C2 domain were identified in PHI group. Twenty-three single peptides (yellow and green), mainly located in the C1/V1/C2/V3 region, were identified in CHI group.

Based on our observation of the ADCC responses to various peptide pools, 20 blood samples from HIV-infected subjects with positive ADCC responses were used to identify single HIV peptides using the matrix formats method. Thirty-three single peptides were identified that provoked ADCC responses in PHI and CHI groups (Tables 2.1, 2.2). Our data showed that 10 single peptides identified in PHI group, were primarily located in the V1/C2 region; while 23 peptides recognized by CHI group, were mainly in the C1/V1/C2/V3 region; and epitopes recognized by both CHI and PHI groups were primarily located in the C1/V1/V2/C2 domain (Figure 5).

**Table 2.2:** Mapping HIV-specific antigen epitopes in CHI group.

**Total responders**	**Peptide no**.	**Sequence**	**Location**
4	5	LWVTVYYGVPVWRDANTT	Env 34 → 51
3	26	TSVIKQACPKISFDPIPI	Env 198 → 215
2	1	MRVKETQMNWPNLWKWGT	Env 1 → 19
2	12	TENFNMWKNNMVEQMQED	Env 90 → 107
2	19	NGIGNITDEVRNCSFNMT	Env 142 → 162
2	29	GYAILKCNDKNFNGTGPC	Env 222 → 239
1	2	NWPNLWKWGTLILGLVMM	Signal peptide
1	3	GTLILGLVMMCSASNNLW	Signal peptide
1	9	HNVWATYACVPTDPNPQE	Env 66 → 83
1	10	CVPTDPNPQEIPLENVTE	Env 74 → 91
1	11	QEIPLENVTENFNMWKNN	Env 82 → 99
1	16	LTPLCVTLNCTNANLTKI	Env 122 → 140
1	17	NCTNANLTKINATtASNG	Env 130 → 145
1	22	KQKVYALFYKLDIVPIID	Env 169 → 185
1	24	IDSSNNSSEYRLINCNTS	Env 184 → 199
1	31	PCKNVSSVQCTHGIKPVV	Env 238 → 255
1	33	VVSTQLLLNGSLAEEEII	Env 254 → 271
1	37	VHLNESVEINCTRPSNNT	Env 286 → 303
1	39	NTRTSINIGPGRAFYRTG	Env 302 → 321
1	41	TGDIIGDIRQAYCEINGT	Env 319 → 336
1	55	APPISGIIKCVSNITGIL	Env 436 → 453
1	57	ILLTRDGGNNNTSETFRP	Env 452 → 470
1	59	RPGGGNIKDNWRSELYKY	Env 469 → 486

**Table 3:** The percentage and magnitude of ADCC response to different peptide pools.

	**Pool 1 (gp120C1V1V2C2)**	**Pool 2 (gp120V3C3V4C4V5)**	**Pool 3 (gp41)**
**ADCC(+) %**			
PHI	83.3	50	50^*^
CHI	77.8	55.6	44.4^*^
HARRT	87.5	56.2	50^*^
**IFN-γ^+^ NK CELLS %**			
PHI	1.83	1.06	1.09
CHI	4.68	1.51	2.16
HAART	3.29	1.47	1.41

* Pool 3 vs. Pool 1 0.05 < p < 0.1

### Evaluation of epitope conservation and spatial conformation

**Figure 6:**
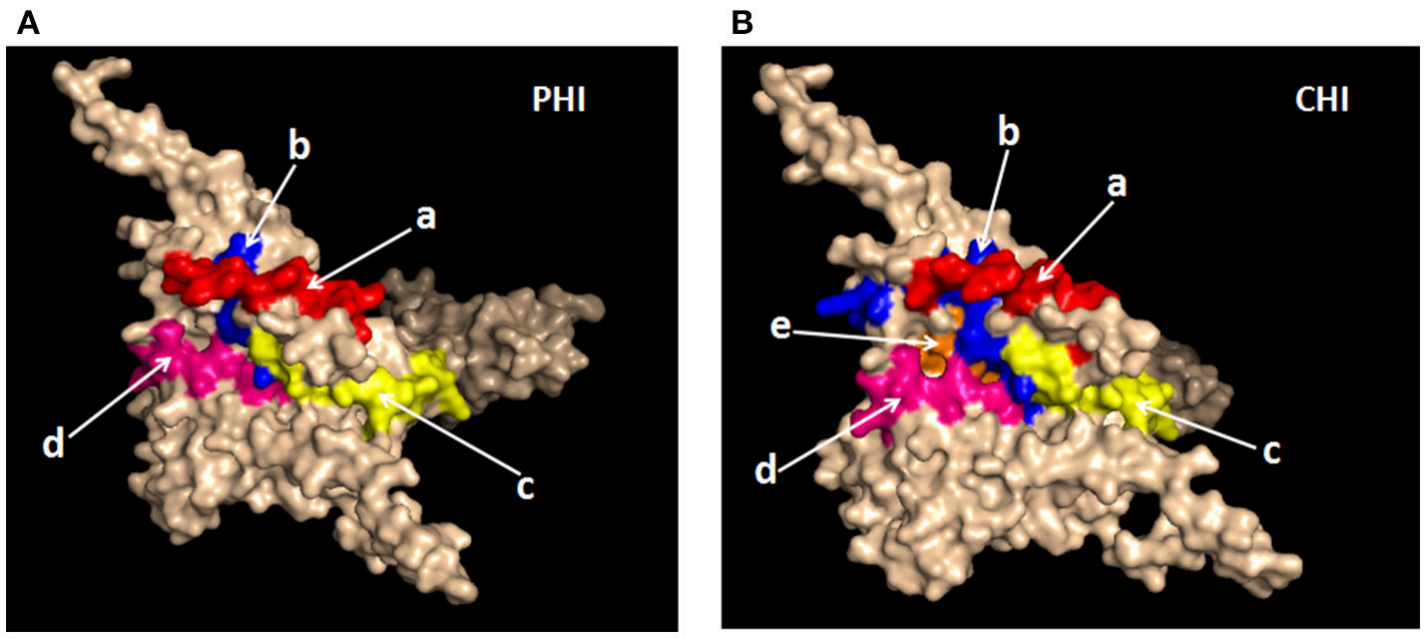
Spatial positions of relatively conserved Env epitopes recognized by ADCC responses in PHI and CHI. **(A)** Simulated spatial structures of the relatively conserved Env epitopes in PHI. a: HNVWATYACVPTDPNPQE (red); b: QCTHGIKPVVSTQLLLNG (blue); c: TSVIKQACPKISFDPIPI (yellow); d: VVSTQLLLNGSLAEEEII (pink). **(B)** Simulated spatial structures of the relatively conserved Env epitopes in CHI. a: HNVWATYACVPTDPNPQE (red); b: PCKNVSSVQCTHGIKPVV (blue); c: TSVIKQACPKISFDPIPI (yellow); d: VVSTQLLLNGSLAEEEII (pink); e: RPGGGNIKDNWRSELYKY (orange).

It was important to determine whether the HIV-specific epitopes of ADCC were conserved and where they mapped in the context of protein spatial structures. Therefore, we calculated epitope prevalence and Shannon entropy scores. Those epitopes with prevalence scores ≥60% and Shannon entropy scores <20th percentile of epitope distribution were defined as conserved epitopes. Furthermore, we constructed spatial structures in SWISS-MODEL, using the Env sequence from one representative local CRF01-AE subtype from our lab. The spatial positions of conserved epitopes are indicated by different colors. There were four conserved epitopes in the PHI group and five in the CHI group, of which three (HNVWATYACVPTDPNPQE, TSVIKQACPKISFDPIPI, and VVSTQLLLNGSLAEEEII), were shared between both groups (Table 4); these three epitopes mapped to the surface of the three-dimensional structure of Env (Figures 6Aacd,Bacd), which may reflect their roles in the ADCC responses of the PHI and CHI groups. These epitopes represent reference sequences potentially useful for to future efforts toward development of antibodies or targeted drugs.

**Table 4:** Relatively conserved epitopes in PHI and CHI groups.

**Sequence**	**Epitope prevalence scores**	**Shannon entropy scores**
**PHI GROUP**		
HNVWATYACVPTDPNPQE	0.5946	0.5807
TSVIKQACPKISFDPIPI	0.5946	0.5915
VVSTQLLLNGSLAEEEII	0.8378	0.5471
QCTHGIKPVVSTQLLLNG	0.9459	0.5287
**CHI GROUP**		
HNVWATYACVPTDPNPQE	0.6	0.59
TSVIKQACPKISFDPIPI	0.6	0.59
VVSTQLLLNGSLAEEEII	0.84	0.53
RPGGGNIKDNWRSELYKY	0.68	0.59
PCKNVSSVQCTHGIKPVV	0.65	0.57

## Discussion

The ADCC response makes an important contribution to antiviral effects and may help to control HIV replication; however, the role of ADCC responses in PHI requires further study. Here, we examined ADCC during PHI and found that 26.19% of individuals in the PHI group exhibited a positive ADCC response, and it was at 52 days after infection that the earliest ADCC response to a single peptide could be defined in our recruited subjects. Our findings also indicated that viral set points were significantly lower in subjects with positive, than in those with negative, ADCC responses. Thus, early ADCC responses may be significant with regard to disease intervention, since they occur early during infection and can both control the viral load and reduce the viral set point. Further support for this viewpoint, stems from the use of HIV-infected primary CD4^+^ T cells as targets, rather than a peptide pool, which were co-cultured with autologous NK cells and serum from individuals with PHI to establish an *in vitro* model of the ADCC response. We initially observed that the ADCC response induced by serum samples from individuals with PHI and the positive ADCC responses were more effective in inhibiting viral infection compared with those from individuals without ADCC responses. There was a slight suppression of HIV infection with NCs' sera, but it was not statistically significant, and the probable reason could be that NCs' sera contains some innate immune components, such as some cytokines (23, 24), which might inhibit viral infection. Moreover, ADCC-mediated viral inhibition could be suppressed using a CD16 blocking antibody. Thus, our results suggest that ADCC responses during PHI are sufficient to control HIV infection and influence the level of the viral set point, thereby influencing disease progression.

Moreover, dynamic changes in the ADCC response during HIV infection, detected using longitudinal follow up samples, have rarely been reported. Our results suggested that the magnitude of the ADCC response within 6 months of HIV infection was relatively low, significantly increasing with continuing infection time, and subsequently decreasing after 2 years. It has been reported that IgG antibodies are involved in the ADCC response (25). In primary HIV infection, the reason for a weak ADCC response might be the low antibody titer and low degree of diversity of the IgG antibodies. After a few months, an increase in the titer and diversity of IgG could result in a stronger ADCC response. Furthermore, our results in this study showed that the ADCC response had a decreased tendency after 2 years of HIV infection but it was not statistically significant. There were two possible reasons. On the one hand, the function of B cells was impaired leading to the decreased secretion of antibodies (26, 27), which caused the decreased ADCC response in the later stages of CHI. On the other hand, there were 40% of viruses that used the CCR5 receptor switching to the CXCR4/DM (dual or mixed) receptor within 3 years of HIV infection (18). The co-receptor switch might have caused the decreased antibody binding, which resulted in the decreased ADCC response in the late stage of CHI. While the specific mechanisms underlying these dynamic changes require further investigation.

Next, we analyzed the percentage and magnitude of ADCC responses induced by different peptide pools. For the majority of ADCC responses, pool 1 of HIV-1 Env was widely recognized and provoked strong reactions in samples from the PHI, CHI, and HAART groups. In addition, epitopes recognized by both CHI and PHI samples were primarily located in the C1/V1/V2/C2 domain. These data indicate that Pool 1 contained the main target for ADCC responses, which could have a role in virus control. Several studies have demonstrated that potent ADCC responses in humans may be directed against Cluster A in the C1–C2 region of the gp120 protein (28–30), which is consistent with our results.

Our data indicated that there were a large number of single peptide epitopes that invoked ADCC responses during CHI. However, in PHI, only a small number of single peptides were identified, which may reflect lower titers and a reduced variety of ADCC antibodies, increasing the difficulty of peptide recognition. Interestingly, among 23 single peptides observed in CHI six, including peptides 5 (LWVTVYYGVPVWRDANTT), 12 (TENFNMWKNNMVEQMQED), 19 (NGIGNITDEVRNCSFNMT), 3 (GTLILGLVMMCSASNNLW), 11 (QEIPLENVTENFNMWKNN), and 21 (MTTLLTDKKQKYYALFYK), were similar to those identified in Thai patients with CHI (subtype HIV-AE) (5). These epitopes may be the most important targets of ADCC antibodies. Other potent ADCC-mediating antibodies have been identified during HIV-1 infection or isolated from RV144 (31, 32); therefore, identification of potential ADCC peptide targets warrants deep exploration. We found three conserved epitopes (VVSTQLLLNGSLAEEEII, HNVWATYACVPTDPNPQE, and TSVIKQACPKISFDPIPI) that stimulated both PHI and CHI samples; importantly, these were located on the surface of the three-dimensional structure of Env. Given the relatively high conservation of these epitopes, they are unlikely to be mutated in the virus; moreover, their locations on the surface of Env facilitate attack of the virus by ADCC antibodies. Hence, these epitopes have potential for use as targets for vaccine design with the aim of virus control.

There was one particular limitation in our study which should be considered when interpreting our results. There is not a single assay able to determine the direct cytotoxic effect, and INF-γ production did not fully reflect cytotoxic behavior in ADCC response. In fact, the ultimate goal of the ADCC response was to inhibit viral infection; according with this, we not only tested the INF-γ production, but also did functional assays about inhibition of viral infection to reflect the ADCC response.

In conclusion, in this study we revealed the importance of ADCC in viral control during PHI and found three conserved epitopes from both PHI and CHI samples that map to the surface of Env and could be applicable in future vaccination studies.

## Author contributions

XC, ML, SQ, YJ, and HS designed the experiments, analyzed the data, and wrote the manuscript. XC, ML, SQ, ZZ, YF, JX, XH, and HD performed the study and conducted experiments. XC, ML, SQ, YJ, and TD analyzed the data. XC, ML, SQ, YJ, and TD wrote and revised the manuscript. All authors read and approved the final manuscript.

### Conflict of interest statement

The authors declare that the research was conducted in the absence of any commercial or financial relationships that could be construed as a potential conflict of interest.
